# Atrial fibrillation per se was a major determinant of global left ventricular longitudinal systolic strain

**DOI:** 10.1097/MD.0000000000004038

**Published:** 2016-07-01

**Authors:** Hung-Hao Lee, Meng-Kuang Lee, Wen-Hsien Lee, Po-Chao Hsu, Chun-Yuan Chu, Chee-Siong Lee, Tsung-Hsien Lin, Wen-Chol Voon, Wen-Ter Lai, Sheng-Hsiung Sheu, Ho-Ming Su

**Affiliations:** a Division of Cardiology, Department of Internal Medicine, Kaohsiung Medical University Hospital; b Department of Internal Medicine, Kaohsiung Municipal Hsiao-Kang Hospital; c Faculty of Medicine, College of Medicine, Kaohsiung Medical University, Kaohsiung, Taiwan.

**Keywords:** atrial fibrillation, global longitudinal systolic strain, left ventricular systolic function

## Abstract

Atrial fibrillation (AF) may cause systolic abnormality via inadequate diastolic filling and tachycardia-induced cardiomyopathy. Global longitudinal strain (GLS) is a very sensitive method for detecting subtle left ventricular systolic dysfunction. Hence, this study aimed to evaluate whether AF patients had a more impaired GLS, AF was a major determinant of GLS, and determine the major correlates of GLS in AF patients.

The study included 137 patients with persistent AF and left ventricular ejection fraction (LVEF) above 50% and 137 non-AF patients matched according to age, gender, and LVEF. Comprehensive echocardiography with GLS assessment was performed for all cases.

Compared with non-AF patients, AF patients had a more impaired GLS, a larger left atrial volume index, higher transmitral E wave velocity (E), and early diastolic mitral velocity (Ea) (all *P* < 0.001) but comparable E/Ea. After adjustment for baseline and echocardiographic characteristics, the presence of AF remained significantly associated with impaired GLS (β = 0.533, *P* < 0.001). In addition, multivariate analysis of AF patients indicated that faster heart rates and decreased E, Ea, and LVEF were associated with more impaired GLS.

This study demonstrated that AF patients had a more impaired GLS than non-AF patients, although LVEF was comparable between the 2 groups. AF was a major determinant of GLS even after adjustment for relevant clinical and echocardiographic parameters.

## 1 Introduction

Atrial fibrillation (AF) is the most commonly sustained arrhythmias in chronic heart failure.^[1]^ AF causes heart failure via 2 possible mechanisms: inadequate diastolic filling and tachycardia-induced cardiomyopathy. When AF evolves, the ventricular filling may be impaired due to loss of active atrial contraction and shortening of the diastolic time, resulting in a 15% to 20% reduction of cardiac output.^[2]^ In addition, paroxysmal tachycardia is frequent in AF patients and may lead to systolic impairment through a mechanism characterized by tachycardia-induced cardiomyopathy, resulting in a further decrease in cardiac output.^[2–4]^ Therefore, AF per se may cause impairment of left ventricular (LV) systolic function.

Evaluation of LV systolic function is a fundamental function and an important indication for echocardiographic examination.^[5,6]^ LV ejection fraction (LVEF) remains the simplest and the most commonly used parameter for assessing LV systolic function. However, there are several technical limitations. Particularly, the quantitative measurements of LVEF require accurate tracing of the endocardial border and the use of formulae that make assumptions about the geometry of LV.^[7]^ Thus, because LVEF denotes global LV function and does not take into account any regional differences, global LV longitudinal systolic strain (GLS) derived from 2-dimensional (2D) speckle-tracking echocardiography has recently emerged as a more sensitive measure of LV systolic function and is used to detect early systolic abnormalities when LVEF is still normal.^[6,8–10]^ This imaging method can discriminate between active and passive myocardial motion and quantify myocardial deformations in 2 dimensions.

Because of beat-to-beat variation, it has always been difficult to estimate LV systolic function in AF. However, our research group and Kusunose et al^[11,12]^ have found that, using the index beat to measure LV longitudinal systolic strain in AF patients is as accurate as averaging multiple cardiac cycles. Consequently, although GLS measured from index beat should be a good systolic parameter in AF patients, no study till date evaluating the influence of AF on GLS has been published. Hence, this study aimed to compare GLS between patients with and without AF in a normal LVEF stage, verify whether AF patients have a more impaired GLS, determine whether AF per se is a major determinant of GLS, and evaluate the major determinants of GLS in AF patients.

## 2 Methods

### 2.1 Study patients

Patients with persistent AF referred for echocardiography between April 1, 2010, and June 30, 2012, at Kaohsiung Municipal Hsiao-Kang Hospital were prospectively and consecutively included in this study. We defined persistent AF as AF that lasted longer than 7 days, which was confirmed by 12-lead electrocardiography (ECG), a 24-hour Holter monitor, or an ECG recording during echocardiographic examination. Patients with moderate and severe mitral stenosis, severe mitral regurgitation, moderate and severe aortic stenosis or regurgitation, LVEF <50%, and inadequate echocardiographic visualization were excluded from the analysis (n = 11). Finally, 137 patients with persistent AF were included in this study. In addition, 137 patients with sinus rhythm who were matched by age, gender, and LVEF and who were referred for echocardiographic examination during the same period were selected as the non-AF patient group. The study protocol was approved by the Institutional Review Board of Kaohsiung Municipal Hsiao-Kang Hospital and written informed consent was obtained from all patients.

### 2.2 Echocardiographic evaluation

Echocardiographic examination was performed using VIVID 7 (General Electric Medical Systems, Horten, Norway) by an experienced cardiologist using a standard protocol, as described in our previous studies.^[13,14]^ The raw ultrasonic data were stored for offline analysis using commercially available software (EchoPAC version 08; General Electric-Vingmed Ultrasound AS, General Electric Medical Systems, Horten, Norway). The cardiologist was blinded to the medical history of all participants, including diabetes mellitus, hypertension, and coronary artery disease. We recorded 2D and M-mode images from standard views, and using the apical 4-chamber view, we obtained LV inflow waveforms and lateral mitral annular tissue Doppler by placing the sample volume at the mitral leaflet tips and lateral annulus, respectively. We measured LVEF using the modified Simpson method, LV mass using the Devereux-modified method,^[15]^ and left atrial volume using the biplane area-length method.^[16]^ LV mass index (LVMI) was calculated by dividing the LV mass by the body surface area. Left atrial volume index (LAVI) was calculated by dividing the left atrial volume by the body surface area.

LV apical 4-chamber, 2-chamber, and long-axis views were acquired using the frame rate between 50 and 90 frames/s. After manually tracing the endocardial border using a point-and-click technique, the system then automatically delineated the epicardial border, thus generating a region of interest cover the entire myocardial wall. A time-strain plot was then automatically produced by the software, and GLS was assessed from the 18 LV segments. Average values were obtained for further analysis, with the minimum acceptable number of LV segments required to perform GLS measurements set to 15.

In AF patients, we measured echocardiographic data using the index beat method.^[11,12,17]^ Because of their simple and fast measurements, the transmitral E-wave velocity (E), E-wave deceleration time (EDT), and early diastolic mitral annulus velocity (Ea) were obtained from 5 beats,^[18]^ and average values were calculated for further analysis. Heart rate was determined using 5 consecutive cardiac cycles. In non-AF patients, echocardiographic data were obtained from 3 consecutive beats to get average values for further analysis.

### 2.3 Demographic, medical, and laboratory data

We obtained demographic and medical data, including age, gender, diabetes mellitus, hypertension, heart failure, cerebrovascular accident, blood pressures, total cholesterol, and triglyceride. Body mass index was calculated as weight in kilograms divided by the square of height in meters. In addition, information regarding each patient's use of medications during the study period was obtained, including angiotensin-converting enzyme inhibitors (ACEIs), angiotensin II receptor blockers (ARBs), β blockers, calcium channel blockers, diuretics, and metformin.

### 2.4 Statistical analysis

SPSS 18.0 software (SPSS, Chicago, IL) was used for all statistical analyses. The data are expressed as mean ± standard deviation or percentage. Dummy coding (one and zero) was used when analyzing categorical variables. Continuous and categorical variables were compared between groups using independent sample *t* tests and Chi-square tests, respectively. The relationship between continuous variables was assessed using a bivariate correlation method (Pearson correlation); if the covariates were significant in the univariate analysis, they were selected for multivariate analysis. A stepwise multiple linear regression analysis was employed to identify the determinants of GLS. All tests were 2-sided, and a *P* value of <0.05 was considered statistically significant.

## 3 Results

Comparisons of clinical and echocardiographic characteristics between patients with and without AF are summarized in Table 1. Mean age of the study population was 67 ± 9 years and 41% were female. There were no differences in age, sex, blood pressure, LVEF, LVMI, and E/Ea between the 2 groups. Compared with non-AF patients, AF patients had a faster heart rate, larger body mass index, higher prevalence of cerebrovascular accident and chronic heart failure, lower total cholesterol, higher LAVI, E, and Ea, lower LV end-diastolic and end-systolic volumes, and EDT, and more impaired GLS.

**Table 1:**
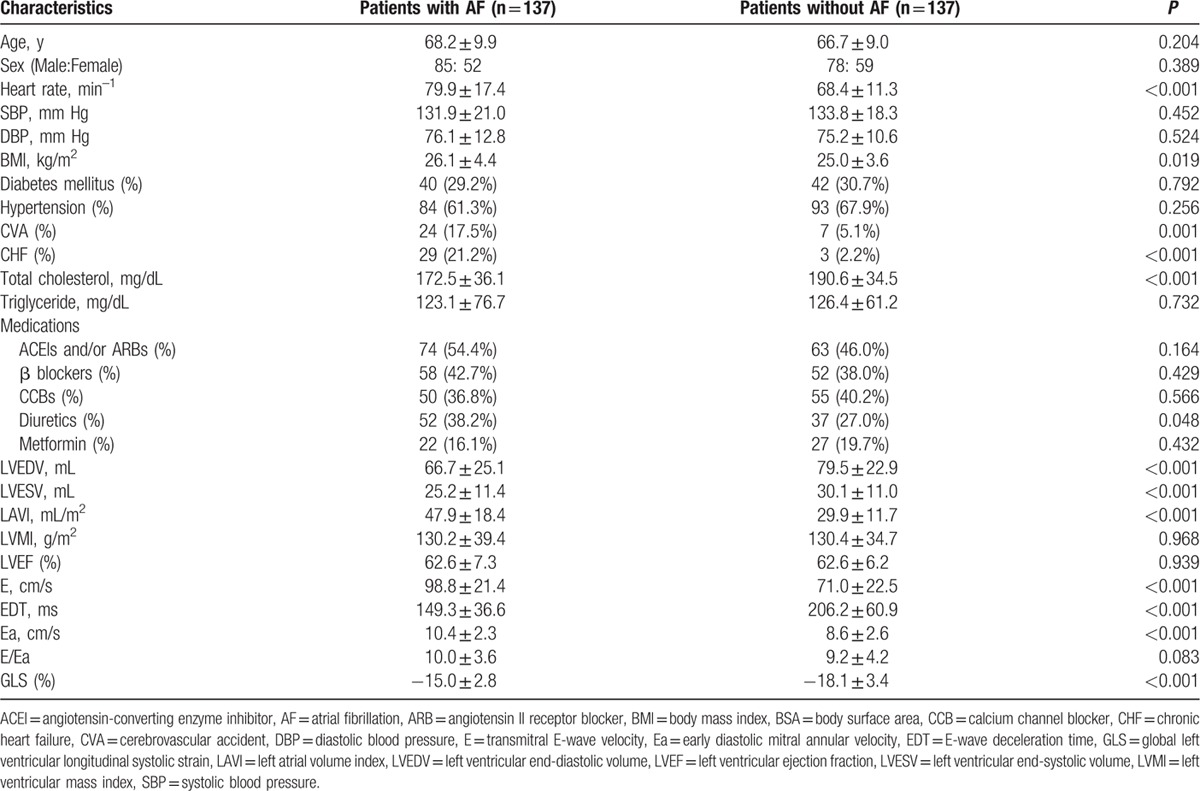
Comparison of clinical and echocardiographic characteristics between patients with and without AF.

Determinants of GLS for all patients are summarized in Table 2. LVEF, EDT, and Ea were negatively associated with GLS, whereas heart rate, blood pressures, AF, diabetes, cerebrovascular accident, chronic heart failure, LAVI, LVMI, and E/Ea were positively associated with GLS in the univariate analysis. Results of the multivariate analysis showed that the presence of AF, faster heart rate, higher diastolic blood pressure and LVMI, lower LVEF, and Ea were associated with more impaired GLS.

**Table 2:**
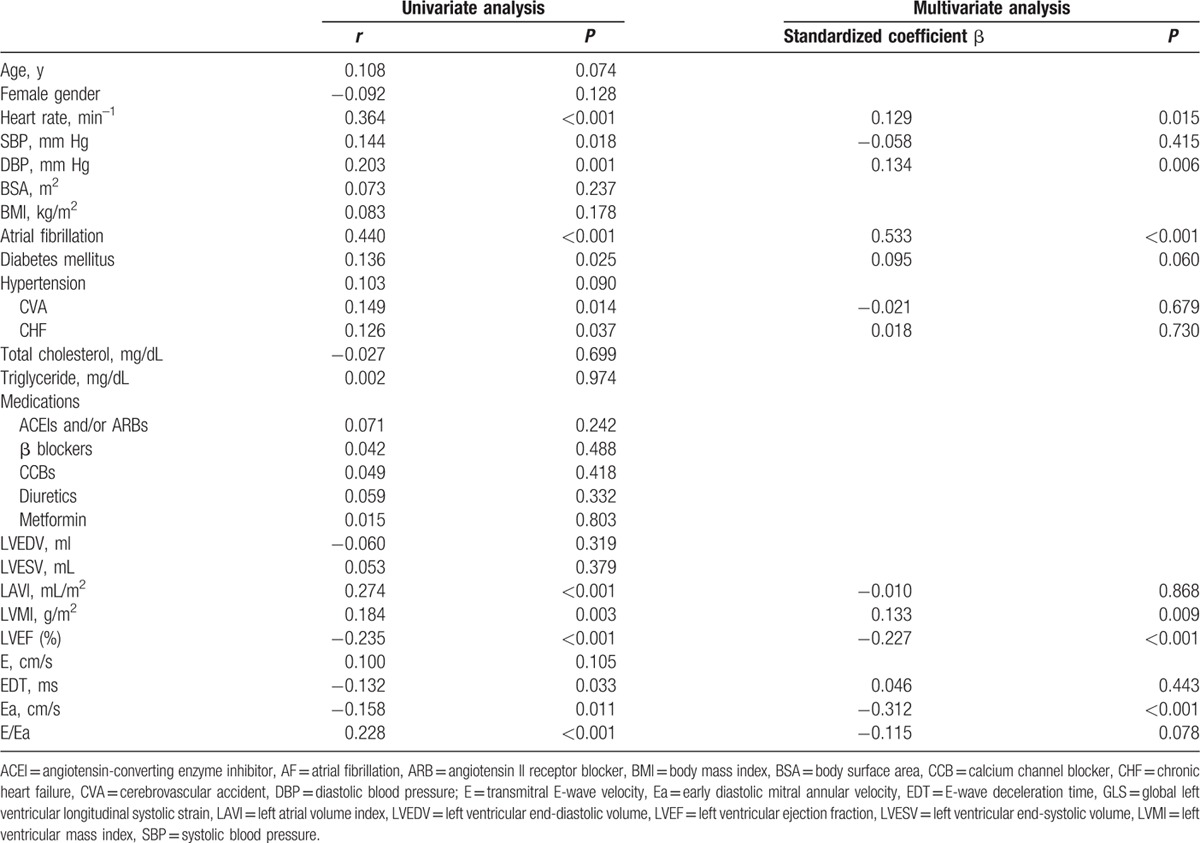
Univariate and multivariate correlates of GLS in all patients.

Determinants of GLS in AF patients are summarized in Table 3. Compared with the data in Table 2, diabetes, cerebrovascular accident, chronic heart failure, LAVI, LVMI, EDT, and E/Ea were not associated with GLS in the univariate analysis, whereas in the multivariate analysis, only heart rate, LVEF, E, and Ea were associated with GLS both in all patients and in AF patients. In addition, LVEF, GLS, Ea, and E/Ea were comparable (*P* ≥ 0.392) between AF patients with and without antihypertensive medications (ACEIs, ARBs, β blockers, calcium channel blockers, and diuretics).

**Table 3:**
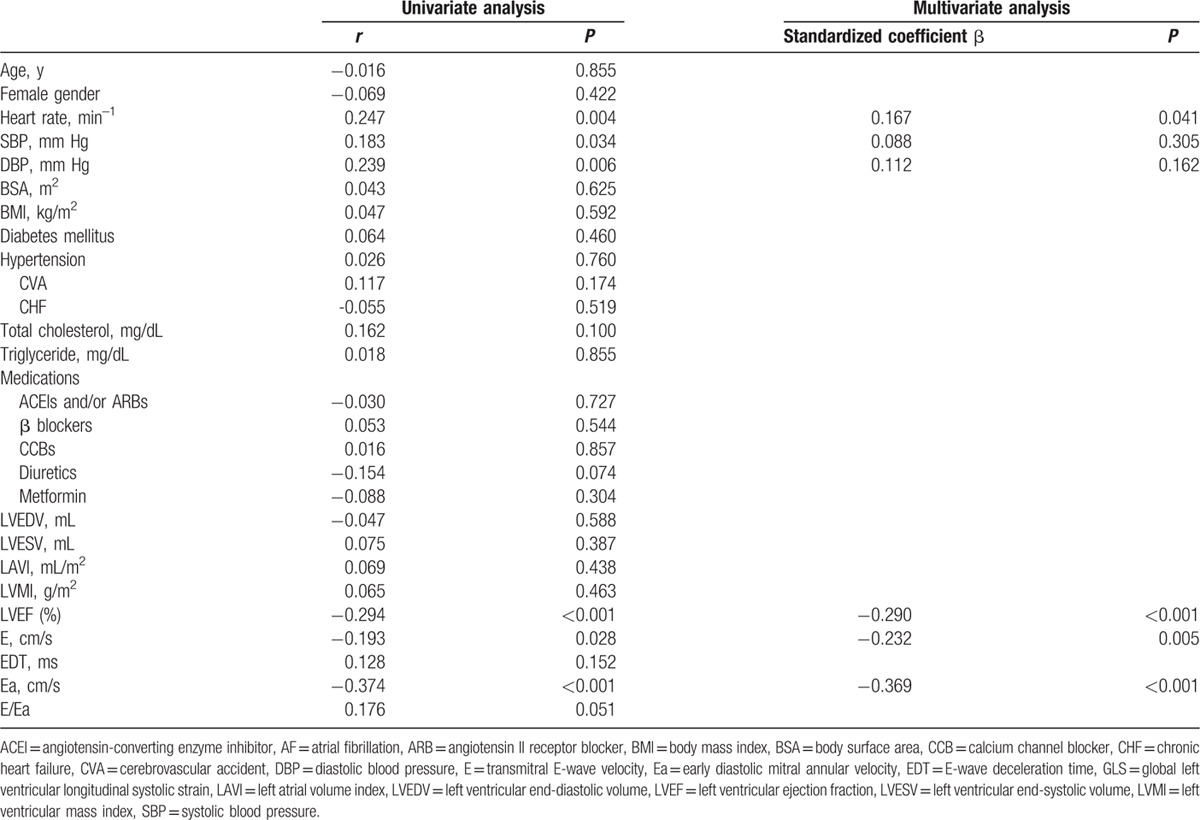
Univariate and multivariate correlates of GLS in patients with AF.

## 4 Discussion

In this study, clinical and echocardiographic parameters were compared between patients with and without AF, alongside determinants of GLS in all patients and in AF patients. Compared with age, gender, and LVEF-matched non-AF patients, AF patients demonstrated significantly impaired GLS. Thus, AF per se was a major determinant of GLS, even after adjustment for baseline and echocardiographic characteristics. Furthermore, heart rate, LVEF, E, and Ea were important determinants of GLS in the AF patients.

AF is characterized by a loss of atrial mechanical contraction, which leads to an inability to enhance LV filling that may compromise hemodynamic performance and cause LV systolic dysfunction.^[2]^ In addition, paroxysmal tachycardia, frequently noted in AF patients, may lead to cardiomyopathy and consequently cause systolic dysfunction.^[19]^ Although insufficient ventricular filling in AF patients can be greatly improved by good rate control, the lack of atrial booster pump function may still impair LV systolic function. Therefore, AF should be an important determinant of LV systolic function. In the present study, even after adjustment for systolic and diastolic blood pressures, heart rate, diabetes, cerebrovascular accident, chronic heart failure, LAVI, LVMI, LVEF, and LV diastolic parameters, AF per se was still a major determinant of GLS.

Using magnetic resonance imaging as the reference standard, Brown et al^[20]^ investigated whether GLS could present an alternative method to measure LVEF in 62 patients with previous infarctions. These authors found that GLS was an efficient method for quantifying global LV function and had a strong correlation with LVEF.^[20]^ In this study, we also found a strong correlation between GLS and LVEF. Similarly, Ea was reported to be a useful parameter for assessing LV diastolic function.^[18,21]^ Galderisi et al^[22]^ reported that GLS was significantly correlated with LV diastolic function. This study has demonstrated that lower Ea was significantly associated with more impaired GLS in all patients, including those with AF.

There is a growing interest in the influence of heart rate on several cardiovascular diseases, such as atherosclerosis, hypertension, myocardial infarction, and heart failure.^[23]^ Heart rate is frequently increased in patients with chronic heart failure and is positively correlated with mortality.^[24,25]^ Heart rate may impact cardiac function; however, compared with a higher heart rate, a lower heart rate may be more beneficial to patients with systolic heart failure.^[26]^ In addition, a clearly inverse relationship exists between heart rate and LV systolic function.^[27]^ In this study, we have consistently revealed herein that faster heart rate was associated with a more impaired GLS.

It is known that AF may impair LV diastolic function through the lack of active atrial contraction.^[28,29]^ However, till date, there has been no work evaluating the influence of AF on GLS, which has recently emerged as a more sensitive measure of early systolic abnormality and a superior predictor of cardiovascular outcome.^[30,31]^ This study found that AF might impair GLS even when LVEF was normal.

### 4.1 Study limitations

There were several limitations of this study. First, in AF patients, many echocardiographic parameters in AF patients were not obtained by averaging multiple cardiac cycles, but were measured using the index beat. Nevertheless, previous studies on AF patients have shown that using the index beat method to measure echocardiographic parameters, including left atrial and LV systolic parameters, was as accurate as the time-consuming method of averaging several cardiac cycles. Second, a majority of the patients in this study were treated chronically with antihypertensive medications, including β blockers in 43% of AF patients and 38% of non-AF patients. For ethical reasons, we did not withdraw these drugs. Therefore, although the effect of antihypertensive drugs on the present findings cannot be excluded, we adjusted the use of drugs in the multivariate analysis. Third, although 2D speckle-tracking echocardiography can generate longitudinal, radial, and circumferential strain measurements and LV twist, only the longitudinal strain was measured and analyzed herein. However, because the subendocardial longitudinal fibers are most vulnerable to several diseases, the longitudinal strain deteriorates earlier than radial and circumferential strains.^[10]^ The longitudinal strain is thus considered to be the most sensitive and reproducible method among various strain measurements.^[32,33]^ Final, because the sample size was small and only patients with persistent AF were included, the findings might be less generalized. In addition, although the retrospective nature of our case–control study was susceptible to the effects of selection bias, we minimized this by using strict selection criteria, consecutively including only those patients with persistent AF. In addition, control patients were selected among those referred for echocardiography during the same period, and all echocardiographic examinations were performed by an experienced cardiologist using the same machine (VIVID 7, General Electric Medical Systems, Horten, Norway).

## 5 Conclusions

This study demonstrated that AF patients had a more impaired GLS than non-AF patients. AF was significantly associated with GLS even after adjustment for relevant clinical and echocardiographic parameters.
